# Development and validation of a predictive model for myocardial injury in children with pneumonia based on interpretable machine learning

**DOI:** 10.3389/fped.2026.1907041

**Published:** 2026-08-27

**Authors:** Shuai Zhang, Jianming Zhang

**Affiliations:** Department of Pediatrics, Fuyang Women’s and Children’s Hospital, Fuyang, Anhui, China

**Keywords:** childhood pneumonia, interaction effect, LASSO regression, myocardial injury, nomogram, restricted cubic spline, risk stratification model

## Abstract

**Objective:**

This study aimed to identify independent factors for pneumonia-related myocardial injury in children, and construct and validate a risk prediction model and stratification system to enable early screening of high-risk patients and guide individualized clinical interventions.

**Methods:**

A total of 222 children hospitalized with pneumonia from January 2023 to May 2026 were enrolled in this retrospective study and equally divided into myocardial injury and non-injury groups (111 cases per group). Admission baseline clinical and laboratory data were collected. Core predictive variables were screened via univariate analysis and LASSO regression. Restricted cubic spline regression was performed to explore the nonlinear relationships between variables and myocardial injury. Multivariate logistic regression was used to identify independent risk and protective factors, while the SHAP algorithm validated variable importance and effect directions. We further analyzed variable interactions and established a nomogram model. Model performance was evaluated using ROC curves, bootstrap calibration, and DCA. Subgroup analysis and a three-tier risk stratification system were conducted for systematic verification.

**Results:**

Univariate analysis screened 11 differential variables, which were compressed to seven core predictors by LASSO regression. Multivariate analysis identified AST (OR = 1.11) and TP (OR = 1.12) as independent risk factors, while serum sodium (OR = 0.81), carbon dioxide combining power (OR = 0.86), and urea (OR = 0.67) were protective factors (all *P* < 0.05), consistent with SHAP validation results. Significant synergistic interaction was found between AST and TP (S = 1.32), whereas AST exerted antagonistic interactions with sodium (S = 0.74) and carbon dioxide combining power (S = 0.78). The nomogram yielded AUCs of 0.902 (training set) and 0.822 (validation set), with calibration errors of 0.021 and 0.035. DCA demonstrated favorable clinical net benefit, and all subgroup AUCs exceeded 0.85. The three-tier stratification system presented significantly different myocardial injury rates (6.12%, 25.36%, and 62.79% for low-, medium-, and high-risk groups, *P* < 0.001).

**Conclusion:**

AST, TP, serum sodium, carbon dioxide combining power, and urea independently affect the risk of pediatric pneumonia-associated myocardial injury with distinct synergistic and antagonistic interactions. The validated nomogram and risk stratification system serve as accurate and stable bedside tools for early risk assessment, facilitating timely clinical intervention and optimized medical resource allocation.

## 1 Introduction

Pneumonia is one of the most prevalent infectious diseases and a leading cause of infectious death in children worldwide, posing a severe threat to global pediatric health. According to Global Burden of Disease 2019 data, lower respiratory tract infections result in approximately 740,000 annual deaths in children under five years old, predominantly attributed to pneumonia (1). In China, pediatric pneumonia ranks first among pediatric hospitalized infectious diseases, accounting for 20%–30% of annual pediatric hospital admissions and consuming massive pediatric medical resources (2, 3). Despite continuous advances in antibiotic and supportive therapies that have improved the overall prognosis of pediatric pneumonia, extrapulmonary organ damage, particularly myocardial injury, remains a common, severe and neglected critical complication that adversely affects children's clinical outcomes (4).

Myocardial injury occurs in 10%–40% of children with pneumonia, with a highly variable incidence depending on patient age, pathogen type and disease severity (5). Complicated myocardial injury significantly increases the risks of arrhythmia, cardiac insufficiency, acute heart failure and even mortality, accompanied by prolonged hospital stays and aggravated disease burden (6). Clinically, the early manifestations of pneumonia-related myocardial injury are insidious and non-specific, making early identification of high-risk children difficult relying solely on clinical symptoms. Although cTnI and CK-MB are classic diagnostic biomarkers for myocardial injury, their limited detection sensitivity, strict sampling timing and procedural constraints restrict their routine and widespread clinical application, especially in primary medical institutions (7, 8). Therefore, developing a convenient, accurate and early risk prediction tool based on routine admission laboratory indicators is an urgent unmet clinical need for the management of pediatric pneumonia.

The occurrence of pneumonia-induced myocardial injury is affected by multiple clinical factors, including systemic inflammatory response, hypoxemia, acid-base and electrolyte disorders, and abnormal liver metabolic function, forming a complex multifactorial pathogenic network (9–13). Such multi-factor interaction characteristics indicate that single indicators are insufficient for early risk judgment, and multidimensional combined indicators are required to construct targeted predictive models for accurate risk stratification.

Traditional multivariate logistic regression is widely used in clinical risk prediction but is limited by multicollinearity susceptibility and subjective variable selection bias when processing high-dimensional clinical variables (14). As visualized predictive tools, nomograms can quantitatively individualize disease risk probability and have been well applied in pediatric critical disease prognosis assessment (15, 16). However, specialized prediction models for pediatric pneumonia complicated with myocardial injury are still scarce, and existing relevant studies have prominent defects such as small sample size, non-standard variable screening and insufficient systematic verification, resulting in poor clinical translational value (17, 18).

Advanced statistical methods and interpretable machine learning algorithms provide new solutions for optimizing clinical prediction models. The LASSO regression can eliminate multicollinearity of high-dimensional variables, screen core predictive indicators objectively and avoid model overfitting via ten-fold cross-validation (19, 20). RCS analysis can further fit the nonlinear dose-response relationship between continuous variables and disease outcomes, compensating for the limitations of traditional linear regression. Furthermore, the SHAP interpretable algorithm effectively solves the “black-box” defect of traditional statistical models, quantifies the importance and action direction of each predictive variable individually, and significantly improves the transparency and clinical credibility of prediction models (21, 22). Combined application of the above methods has achieved excellent predictive performance in organ dysfunction prediction and critical pediatric disease prognosis (23, 24).

In view of the deficiencies of current studies in variable selection standardization, model interpretability and systematic validity verification, this study adopted real-world pediatric pneumonia clinical data, integrated LASSO regression, RCS nonlinear analysis, multivariate logistic regression and SHAP interpretable machine learning framework to develop and validate a targeted nomogram prediction model for myocardial injury in children with pneumonia. We further analyzed the interaction effects among core risk factors and established a three-tier clinical risk stratification system. This study aims to provide an accurate, stable and clinically operable tool for early bedside risk identification and individualized clinical intervention for children at high risk of myocardial injury secondary to pneumonia.

## 2 Materials and methods

### 2.1 Study population

**Figure 1:**
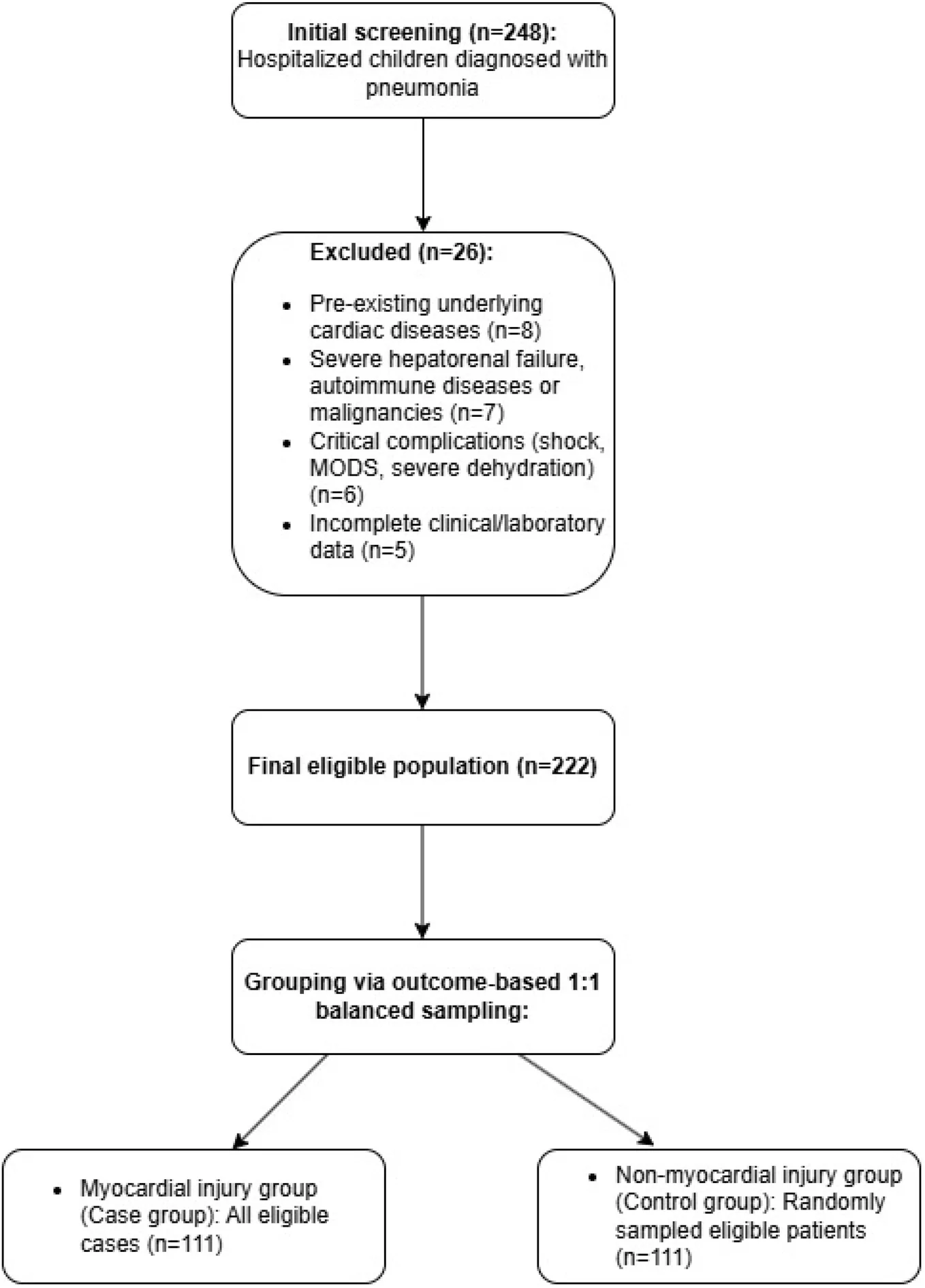
Participant enrollment flowchart of the retrospective cohort study. Flow diagram illustrates the process of initial patient screening, exclusion based on predefined eligibility criteria, and final outcome-based 1:1 balanced grouping. A total of 248 hospitalized children with pneumonia were initially screened, and 26 patients were excluded due to pre-existing cardiac diseases, severe comorbidities, critical complications, or incomplete data. The final enrolled cohort consisted of 111 patients with pneumonia complicated with myocardial injury (case group, fully enrolled) and 111 randomly selected eligible patients with uncomplicated pneumonia (control group), yielding a balanced sample size for subsequent statistical analysis.

This retrospective study enrolled pediatric patients diagnosed with pneumonia who were hospitalized in the Department of Pediatrics at our hospital from January 2023 to May 2026.A complete participant enrollment flow diagram was supplemented in this study following the STROBE guidelines to clearly illustrate patient screening, exclusion, and grouping procedures. During the study period, a total of 248 children with pneumonia were initially screened. Among them, 26 patients were excluded due to pre-existing cardiac diseases, severe organ dysfunction, malignant diseases, critical complications, or incomplete clinical and laboratory data. Ultimately, a total of 222 eligible patients were included in the final analysis (Figure 1). This study adopted an outcome-based 1:1 balanced sampling design. All eligible children with pneumonia complicated with myocardial injury during the study period were fully included as the case group (111 cases). Meanwhile, 111 eligible children with uncomplicated pneumonia without myocardial injury were randomly sampled from the qualified non-injury population as the control group, forming a total study cohort of 222 patients with equal group sample sizes. No individual pairwise matching was performed between the two groups, and all enrolled subjects unifiedly followed the strict inclusion and exclusion criteria. Inclusion criteria were as follows: (1) diagnosis of childhood pneumonia in accordance with the Guidelines for the Diagnosis and Treatment of Community-Acquired Pneumonia in Children; (2) age ranging from 1 month to 14 years; (3) complete baseline laboratory data available at admission, including routine blood parameters, inflammatory biomarkers, electrolytes, hepatic and renal function, and metabolic profiles; (4) first hospitalization for pneumonia without prior targeted interventions, such as myocardial nutrition therapy or electrolyte correction, before admission. Exclusion criteria included the presence of any of the following: (1) underlying cardiac diseases, including congenital heart malformations, previous viral myocarditis, or chronic heart failure; (2) concomitant severe hepatic or renal insufficiency, autoimmune diseases, hematological disorders, or malignant tumors; (3) critical complications such as shock, severe dehydration, or multiple organ dysfunction syndrome; (4) incomplete clinical or laboratory data that hindered statistical analysis. This study was approved by the Medical Ethics Committee of our hospital. Written informed consent was waived due to the retrospective nature of the study.

### 2.2 Grouping criteria

All enrolled patients were divided into myocardial injury and non-myocardial injury groups according to the presence or absence of in-hospital myocardial injury. The diagnosis of myocardial injury was established in strict accordance with the Expert Consensus on the Diagnosis of Pediatric Myocardial Injury (25), and patients who met any of the following conditions were diagnosed with myocardial injury: (1) serum cardiac troponin I (cTnI) level exceeding the upper laboratory reference limit; (2) creatine kinase-MB (CK-MB) activity ≥ 2-fold of the upper laboratory reference value, lactate dehydrogenase (LDH) > 250 U/L, or hydroxybutyrate dehydrogenase (HBDH) > 182 U/L; (3) newly onset electrocardiographic abnormalities, including ST-T segment changes, ectopic rhythms, tachycardia, or bradycardia; (4) echocardiographic manifestations of decreased myocardial motility or mild ventricular dysfunction.

### 2.3 Data collection

Baseline demographic characteristics and admission laboratory data were collected systematically, covering multiple categories of clinical indicators: (1) demographic and hospitalization information: age, sex, body weight, and length of hospital stay; (2) inflammatory biomarkers: procalcitonin (PCT), C-reactive protein (CRP), white blood cell (WBC) count, and neutrophil percentage; (3) electrolyte and acid-base balance parameters: serum potassium, sodium, chloride, and carbon dioxide combining power; (4) hepatic and renal function as well as bilirubin metabolic indices: aspartate aminotransferase (AST), alanine aminotransferase (ALT), albumin (ALB), total protein (TP), alkaline phosphatase (ALP), gamma-glutamyl transferase (GGT), total bilirubin, direct bilirubin, indirect bilirubin, and total bile acids; (5) metabolic and renal function markers: creatinine, urea, uric acid (UA), and fasting plasma glucose (FPG). All laboratory measurements were conducted by the hospital's Clinical Laboratory Department in strict accordance with standardized operating procedures and quality control guidelines.

### 2.4 Statistical methods

All statistical analyses were performed using R software (version 4.3.0). For continuous variables, data conforming to a normal distribution were presented as mean ± standard deviation (SD) and compared between groups via independent sample t-tests, while non-normally distributed data were expressed as median (interquartile range, Q1–Q3) and analyzed using the Wilcoxon rank-sum test. Categorical variables were summarized as counts and percentages [n (%)] and compared using the Chi-square test or Fisher's exact test, as appropriate. A two-tailed *P* value < 0.05 was defined as statistically significant.

Variance inflation factor (VIF) was calculated to evaluate multicollinearity among candidate variables, and variables with VIF > 10 were considered to have severe collinearity and excluded. All baseline variables with significant intergroup differences were enrolled in the least absolute shrinkage and selection operator (LASSO) regression model. The optimal regularization parameter *λ* was determined by 10-fold cross-validation. This approach zeroed the coefficients of redundant and low-contribution variables, effectively eliminating multicollinearity and avoiding subjective bias inherent in conventional variable screening strategies.

Restricted cubic spline (RCS) regression was applied to explore nonlinear dose-response relationships between LASSO-screened continuous variables and myocardial injury, and to identify optimal risk threshold values. Interaction terms were constructed for core predictors, and both multiplicative and additive interaction analyses were conducted. Additive interaction parameters, including the relative excess risk due to interaction (RERI), attributable proportion (AP), and synergy index (S), were calculated to quantify potential synergistic or antagonistic effects between variables.

Multivariate logistic regression was further performed to identify independent risk and protective factors for myocardial injury based on the LASSO-derived variables. The SHapley Additive exPlanations (SHAP) algorithm was adopted to validate variable importance and predictive direction. The mean absolute SHAP value quantified the contribution of each predictor, and SHAP summary plots visualized the magnitude and direction of variable effects, which strengthened the robustness of the regression results. Independent predictors were finally integrated to construct a predictive nomogram model. Model discrimination was assessed using receiver operating characteristic (ROC) curves and the corresponding area under the curve (AUC). Calibration performance was validated via 1000 bootstrap resamples, with the mean absolute error between predicted and observed risks calculated quantitatively. Decision curve analysis (DCA) was performed to evaluate the clinical net benefit and practical applicability of the nomogram.

Stratified subgroup analyses based on age, sex, and disease course were conducted to verify the model's predictive stability across diverse populations. According to the nomogram scoring system, patients were divided into low-, medium-, and high-risk subgroups, and the intergroup differences in myocardial injury incidence were compared to validate the efficacy of risk stratification. All statistical tests were two-tailed, and *P* < 0.05 indicated statistical significance.

## 3 Results

### 3.1 Univariate analysis of baseline characteristics

A total of 222 pediatric patients were enrolled in this study, with 111 cases allocated to the myocardial injury group and another 111 to the non-myocardial injury group. Baseline clinical characteristics of the two groups are summarized in Table 1. No significant intergroup difference was observed in gender distribution (*P* = 0.282). Patients with myocardial injury presented significantly younger age and lower body weight than those without myocardial injury (*P* = 0.003 and *P* = 0.009, respectively), whereas no remarkable difference was found in the length of hospital stay (*P* = 0.628). In terms of electrolytes and renal function, the myocardial injury group had higher serum potassium levels and lower levels of chloride, sodium, carbon dioxide combining power, creatinine, and urea, with all differences reaching statistical significance (all *P* < 0.05). Serum albumin levels were comparable between the two groups (*P* = 0.517). For hepatic function indicators, the myocardial injury group showed significantly elevated aspartate aminotransferase (AST), alanine aminotransferase (ALT), and total protein (TP) levels (all *P* < 0.05). In contrast, no significant differences were detected in total bilirubin, direct bilirubin, indirect bilirubin, total bile acids, alkaline phosphatase (ALP), and gamma-glutamyl transferase (GGT) levels (all *P* > 0.05). Additionally, no statistically significant intergroup differences were found in inflammatory markers (procalcitonin [PCT], C-reactive protein [CRP]), routine blood parameters (neutrophil percentage, lymphocyte percentage, hemoglobin, white blood cell count, platelet count), uric acid, and fasting blood glucose (all *P* > 0.05).

**Table 1:** Univariate analysis of baseline characteristics.

Variables	Total (*n* = 222)	Non-Myocardial damage(*n* = 111)	Myocardial damage (*n* = 111)	Statistic	*P*
Potassium (mmol/L), Mean ± SD	4.58 ± 0.64	4.40 ± 0.57	4.77 ± 0.66	t = −4.57	<0.001
Chlorine (mmol/L), Mean ± SD	103.70 ± 3.32	104.40 ± 3.12	102.99 ± 3.38	t = 3.24	0.001
Carbon Dioxide (mmol/L), Mean ± SD	22.10 ± 3.21	23.16 ± 2.94	21.03 ± 3.14	t = 5.22	<0.001
ALB (g/L), Mean ± SD	42.35 ± 3.35	42.20 ± 3.56	42.49 ± 3.12	t = −0.65	0.517
Creatinine (*μ*mol/L), Mean ± SD	25.76 ± 10.20	28.78 ± 11.26	22.74 ± 7.98	t = 4.62	<0.001
Urea (mmol/L), Mean ± SD	3.41 ± 1.05	3.63 ± 1.07	3.19 ± 0.99	t = 3.18	0.002
Neutrophil percentage (%), Mean ± SD	48.88 ± 18.48	50.78 ± 17.91	46.97 ± 18.92	t = 1.54	0.125
Lymphocyte percentage (%), Mean ± SD	41.93 ± 17.54	39.78 ± 16.64	44.08 ± 18.21	t = −1.84	0.067
Hemoglobin (g/L), Mean ± SD	119.59 ± 10.69	118.62 ± 10.86	120.56 ± 10.47	t = −1.35	0.178
Age (years), M (Q₁, Q₃)	2.15 (1.00, 3.00)	2.60 (1.30, 5.00)	1.80 (0.90, 3.00)	Z = −2.99	0.003
Body weight (Kg), M (Q₁, Q₃)	12.00 (10.00, 17.00)	13.50 (10.25, 20.75)	11.50 (10.00, 15.00)	Z = −2.62	0.009
Length of stay (d), M (Q₁, Q₃)	6.00 (5.00, 7.00)	6.00 (5.00, 7.00)	6.00 (5.00, 7.00)	Z = −0.49	0.628
PCT (ng/mL), M (Q₁, Q₃)	0.08 (0.05, 0.14)	0.08 (0.05, 0.13)	0.08 (0.05, 0.16)	Z = −0.82	0.411
CRP (mg/mL), M (Q₁, Q₃)	6.08 (2.00, 14.37)	6.42 (2.00, 14.55)	5.35 (1.97, 14.36)	Z = −0.58	0.565
Sodium (mmol/L), M (Q₁, Q₃)	138.15 (136.60, 139.78)	138.80 (137.50, 140.45)	137.70 (135.60, 139.35)	Z = −3.77	<0.001
Tota bilirubin (μmol/L), M (Q₁, Q₃)	5.60 (4.30, 7.10)	5.20 (4.00, 7.00)	5.80 (4.80, 7.10)	Z = −1.26	0.209
Direct bilirubin (μmol/L), M (Q₁, Q₃)	2.20 (1.50, 2.80)	2.00 (1.30, 2.70)	2.30 (1.52, 2.98)	Z = −1.45	0.146
Indirect bilirubin (μmol/L), M (Q₁, Q₃)	3.40 (2.50, 4.60)	3.40 (2.50, 4.60)	3.40 (2.60, 4.60)	Z = −0.10	0.919
Total bile acids (μmol/L), M (Q₁, Q₃)	4.90 (2.65, 7.38)	4.90 (2.45, 7.15)	4.90 (2.80, 7.95)	Z = −0.78	0.433
AST (U/L), M (Q₁, Q₃)	43.00 (34.60, 52.20)	36.00 (26.75, 43.40)	49.90 (43.00, 58.35)	Z = −8.13	<0.001
ALT (U/L), M (Q₁, Q₃)	17.75 (14.00, 24.93)	15.50 (12.15, 21.10)	21.00 (16.95, 26.90)	Z = −4.98	<0.001
TP (g/L), M (Q₁, Q₃)	68.20 (65.10, 71.80)	67.40 (64.35, 71.05)	69.50 (66.00, 72.25)	Z = −2.49	0.013
ALP (U/L), M (Q₁, Q₃)	197.45 (162.88, 232.65)	197.90 (162.65, 229.90)	197.00 (164.05, 241.70)	Z = −0.26	0.798
GGT (U/L), M (Q₁, Q₃)	13.40 (11.00, 16.28)	13.50 (11.00, 17.00)	13.40 (11.30, 16.10)	Z = −0.31	0.757
UA (μmol/L), M (Q₁, Q₃)	228.70 (180.40, 278.70)	231.40 (190.30, 278.85)	228.35 (164.50, 274.42)	Z = −0.82	0.413
FPG (μmol/L), M (Q₁, Q₃)	4.87 (4.17, 5.64)	4.84 (4.21, 5.61)	4.91 (4.12, 5.74)	Z = −0.17	0.868
WBC (×10⁹/L), M (Q₁, Q₃)	8.80 (6.57, 11.92)	8.78 (6.79, 11.47)	8.82 (6.46, 12.02)	Z = −0.07	0.941
Platelets (×10⁹/L), M (Q₁, Q₃)	287.50 (227.25, 346.00)	293.00 (246.50, 352.50)	284.00 (212.50, 345.50)	Z = −1.18	0.238
Gender, *n* (%)				*χ*^2^ = 1.16	0.282
Female	104 (46.85)	48 (43.24)	56 (50.45)		
Male	118 (53.15)	63 (56.76)	55 (49.55)		

t, *t*-test; Z, Mann–Whitney test; χ^2^, Chi-square test; SD, standard deviation; M, median; Q_1_, 1st quartile; Q_3_, 3st quartile; PCT, procalcitonin; CRP, C-reactive protein; WBC, white blood cell count; AST, aspartate aminotransferase; ALT, alanine aminotransferase; ALB, albumin; TP, total protein; ALP, alkaline phosphatase; GGT, gamma-glutamyl transferase; UA, uric acid; FPG, fasting plasma glucose.

### 3.2 Variable selection by LASSO regression

**Figure 2:**
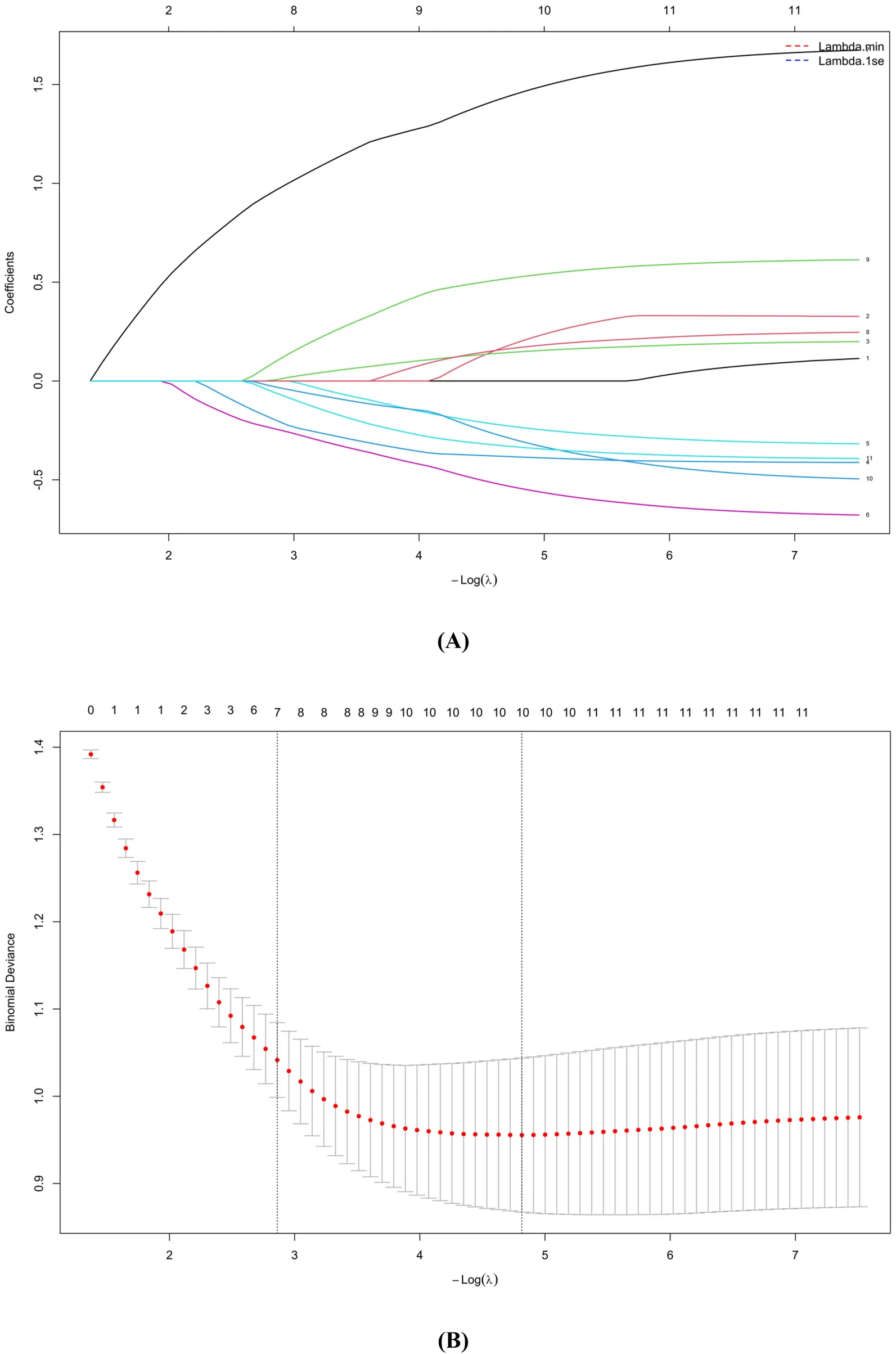
Variable selection via LASSO regression. **(A)** (Coefficient trajectory plot): with the change of *λ* value, the coefficients of all variables are gradually shrunk toward zero, and finally five core indicators with non-zero coefficients are retained; **(B)** (10-fold cross-validation plot): the optimal *λ* value is determined based on the minimum mean square error to screen the optimal variable combination.

All variables with significant intergroup differences in univariate analysis were incorporated into the least absolute shrinkage and selection operator (LASSO) regression model. The optimal regularization parameter *λ* was identified via 10-fold cross-validation, and seven non-redundant core predictors were finally screened out: serum potassium, sodium, carbon dioxide combining power, creatinine, urea, AST, and total protein. Variables including age, body weight, chloride, and ALT were excluded due to low predictive contribution and redundant correlations. This procedure effectively eliminated multicollinearity and minimized subjective selection bias, thereby establishing an optimized predictor panel for model construction. The LASSO coefficient trajectories and cross-validation error curves are displayed in Figure 2.

### 3.3 Nonlinear threshold analysis via restricted cubic spline (RCS)

**Figure 3:**
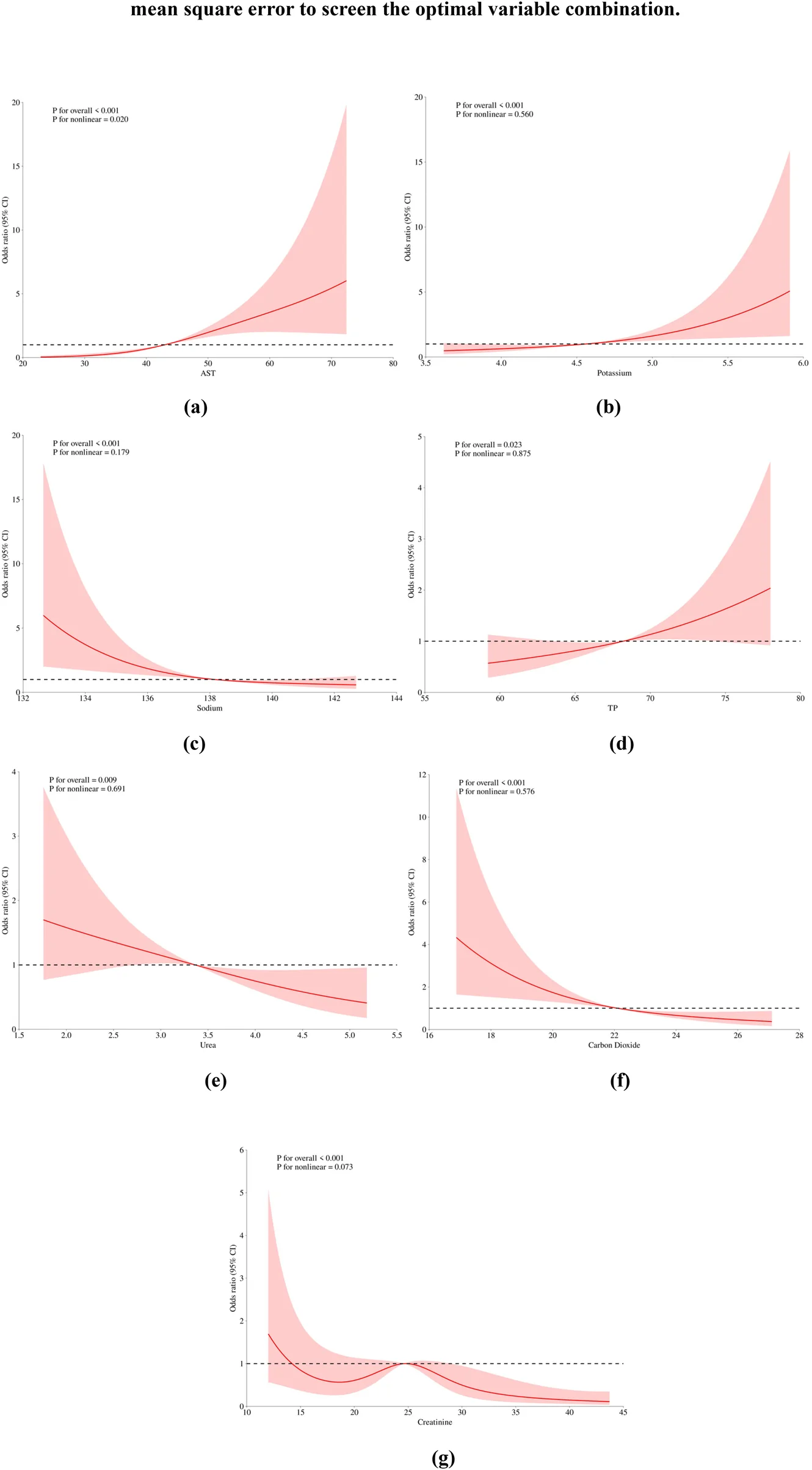
Non-linear dose-response curves of core indicators fitted by restricted cubic splines (RCS). The solid red lines represent the fitted risk values, and the red shaded areas indicate the 95% confidence intervals. The horizontal axis denotes the level of each indicator, and the vertical axis represents the incidence probability of myocardial injury. The plots visually illustrate the threshold mutation effect of each indicator. **(a)** Aspartate aminotransferase; **(b)** Serum potassium; **(c)** Serum sodium; **(d)** Total protein; **(e)** Urea; **(f)** Carbon dioxide combining power; **(g)** Creatinine. The solid lines stand for the fitted risk curves, the shaded areas represent 95% confidence intervals, and the vertical dashed lines mark the risk thresholds.

Restricted cubic spline (RCS) regression was performed to evaluate the nonlinear dose-response relationships between the screened predictors and myocardial injury risk after adjusting for confounding factors (Figure 3). AST exhibited a significant nonlinear correlation with myocardial injury (overall *P* < 0.001, nonlinear *P* = 0.020), with disease risk remaining stable below 45 U/L and rising continuously beyond this threshold. No significant nonlinear trends were observed for serum potassium, sodium, total protein, urea, or carbon dioxide combining power (nonlinear *P*-values: 0.560, 0.179, 0.875, 0.691, 0.576, respectively; all overall *P* < 0.05). Specifically, the risk of myocardial injury increased linearly when serum potassium exceeded 4.6 mmol/L and total protein exceeded 68 g/L. In contrast, decreased levels of sodium (< 138 mmol/L), urea (< 3.4 mmol/L), and carbon dioxide combining power (< 22 mmol/L) were associated with a linear increase in risk, which plateaued once these indicators surpassed the corresponding thresholds. Creatinine showed a borderline nonlinear association (nonlinear *P* = 0.073, overall *P* < 0.001) with a J-shaped distribution: elevated risk was observed at creatinine levels below 18 μmol/L, while relatively low and stable risk was maintained within 18–25 μmol/L, with no further obvious fluctuations at higher concentrations.

### 3.4 Multivariate logistic regression and variable importance validation

**Figure 4:**
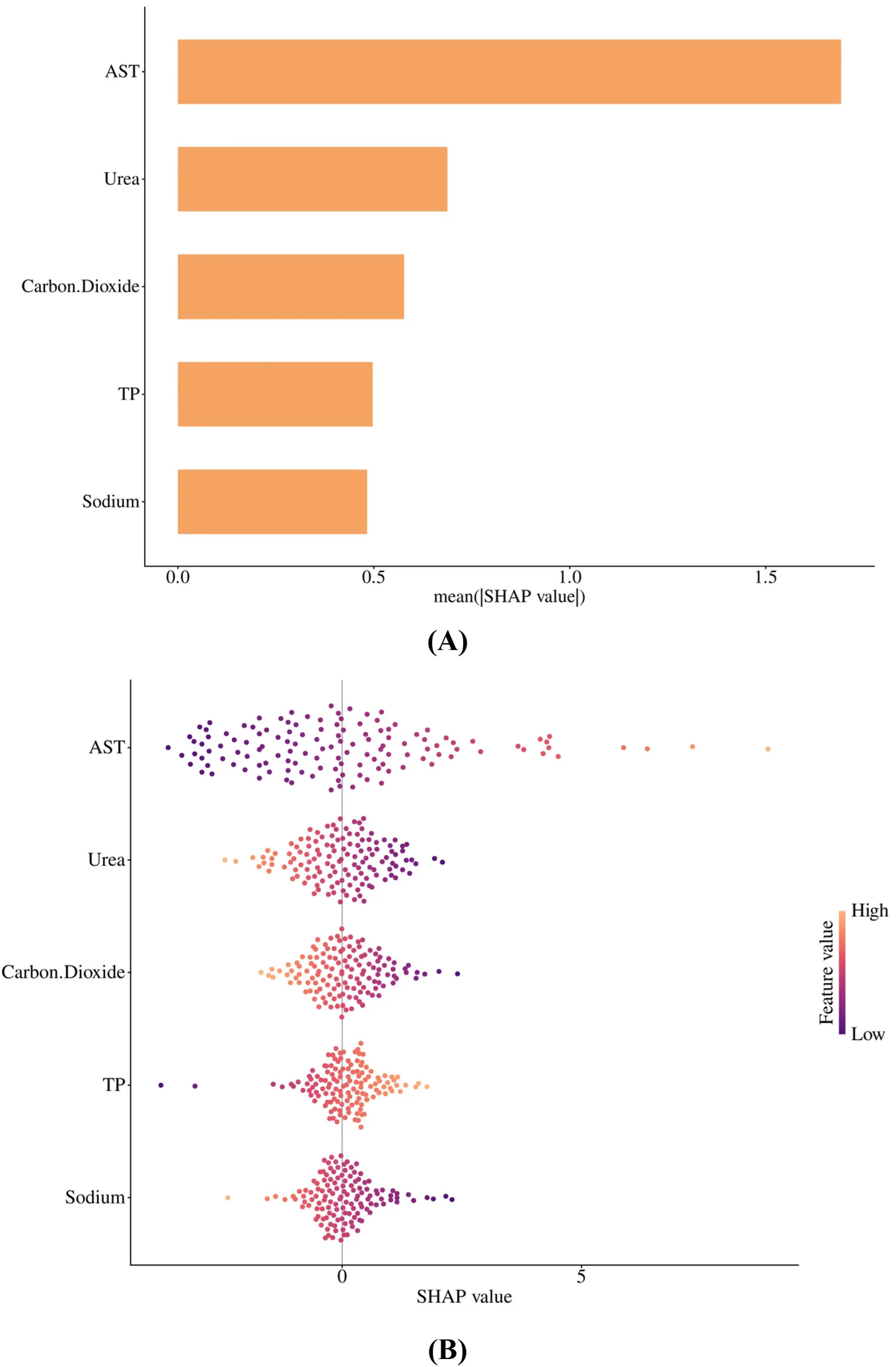
SHAP analysis for variable importance and effect direction. **(A)** Variable importance ranking plot: The length of each bar represents the mean absolute SHAP value of the corresponding variable, indicating its contribution to model prediction; **(B)** SHAP summary scatter plot: Each dot stands for an individual sample. The color gradient ranges from low variable values (purple) to high variable values (orange). The horizontal axis shows SHAP values; positive values correlate with an increased risk of the outcome, while negative values correlate with a decreased risk.

Multivariate logistic regression analysis was performed to identify independent predictors of myocardial injury (Table 2). The results demonstrated that AST (OR = 1.11, 95% CI: 1.07–1.15, *P* < 0.001) and total protein (TP) (OR = 1.12, 95% CI: 1.04–1.21, *P* = 0.003) were independent risk factors for myocardial injury in children with pneumonia. In contrast, serum sodium (OR = 0.81, 95% CI: 0.70–0.93, *P* = 0.003), carbon dioxide combining power (OR = 0.86, 95% CI: 0.76–0.97, *P* = 0.014), and urea (OR = 0.67, 95% CI: 0.45–0.99, *P* = 0.043) acted as independent protective factors. By comparison, creatinine (OR = 0.97, 95% CI: 0.93–1.02, *P* = 0.270) and serum potassium (OR = 1.33, 95% CI: 0.70–2.54, *P* = 0.384) exhibited no significant correlation with myocardial injury risk. SHapley Additive exPlanations (SHAP) analysis was further applied to validate the importance and directional effects of each predictor (Figure 4). The rank of mean absolute SHAP values verified AST as the most influential predictor, followed by urea, carbon dioxide combining power, TP, and sodium, which was highly consistent with the regression outcomes. SHAP summary plots indicated that elevated AST and TP levels corresponded to increased myocardial injury risk (positive SHAP values), while higher levels of sodium, carbon dioxide combining power, and urea were associated with reduced disease risk (negative SHAP values). These findings further confirmed the reliability of the multivariate logistic regression results.

**Table 2:** Multivariate logistic regression analysis of independent risk factors.

Variables	*β*	*S.E*	*Z*	*P*	OR (95%CI)
AST	0.10	0.02	5.40	<0.001	1.11 (1.07–1.15)
Carbon Dioxide	−0.16	0.06	−2.44	0.014	0.86 (0.76–0.97)
Sodium	−0.22	0.07	−3.01	0.003	0.81 (0.70–0.93)
TP	0.11	0.04	3.01	0.003	1.12 (1.04–1.21)
Creatinine	−0.03	0.02	−1.10	0.270	0.97 (0.93–1.02)
Urea	−0.41	0.20	−2.02	0.043	0.67 (0.45–0.99)
Potassium	0.29	0.33	0.87	0.384	1.33 (0.70–2.54)

OR, odds ratio; CI, confidence interval; AST, aspartate aminotransferase; TP, total protein.

### 3.5 Interaction analysis

Based on the logistic regression results, the five independent predictors were categorized into risk factors (AST and TP) and protective factors (sodium, carbon dioxide combining power, and urea). Pairwise multiplicative and additive interaction analyses were subsequently conducted within and across these two groups (Table 3). Multiplicative interaction analysis revealed significant interactions between AST and TP within the risk factor group (*P* = 0.018), as well as cross-group interactions between AST and sodium (*P* = 0.032) and between AST and carbon dioxide combining power (*P* = 0.027). All other variable pairs showed no significant multiplicative interactions (all *P* > 0.05). Additive interaction analysis was performed using bootstrap-derived indicators, including the relative excess risk due to interaction (RERI), attributable proportion (AP), and synergy index (S), with corresponding 95% confidence intervals (CIs). The results were as follows. For AST and TP: RERI = 0.29 (95% CI: 0.08–0.49), AP = 0.16 (95% CI: 0.05–0.27), *S* = 1.32 (95% CI: 1.06–1.64), confirming a significant additive synergistic interaction that accounted for 16% of the total myocardial injury risk. For AST and sodium: RERI = −0.21 (95% CI: −0.39 to −0.04), AP = –0.13 (95% CI: −0.24 to −0.02), *S* = 0.74 (95% CI: 0.51–0.96). For AST and carbon dioxide combining power: RERI = −0.18 (95% CI: −0.35 to −0.02), AP = –0.11 (95% CI: −0.21 to −0.01), *S* = 0.78 (95% CI: 0.56–0.98). The latter two pairs presented significant antagonistic interactions (*S* < 1). No significant additive interactions were observed for variable pairs without significant multiplicative interactions.

**Table 3:** Analysis of double interaction effects.

Interaction combination type	Interactive combination	Multiplicative interaction:Beta (SE)	Z-value	*P*-value	Additive interaction: RERI (95% CI)	Additive interaction: AP(95% CI)	Additive interaction:S (95% CI)	Type of Effect
Risk Factor Group Combined								
AST*TP		0.021 (0.009)	2.333	0.018	0.29 (0.08–0.49)	0.16 (0.05–0.27)	1.32 (1.06–1.64)	Significant synergistic effect
Within group combinations of protective factors								
Serum sodium*Carbon dioxide		−0.018 (0.011)	−1.636	0.102	−	−	−	No significant interaction
Serum sodium*Urea		−0.009 (0.013)	−0.692	0.489	−	−	−	No significant interaction
Carbon dioxide*Urea		−0.012 (0.015)	−0.800	0.424	−	−	−	No significant interaction
Cross combination between groups								
AST*Serum sodium		−0.015 (0.007)	−2.143	0.032	−0.21(−0.39 to −0.04)	−0.13(−0.24 to −0.02)	0.74 (0.51–0.96)	Significant antagonistic effect
AST*Carbon dioxide		−0.013 (0.006)	−2.217	0.027	−0.18(−0.35 to −0.02)	−0.11(−0.21 to −0.01)	0.78 (0.56–0.98)	Significant antagonistic effect
AST*Urea		−0.008 (0.009)	−0.889	0.374	−	−	−	No significant interaction
TP*Serum sodium		−0.011 (0.008)	−1.375	0.169	−	−	−	No significant interaction
TP*Carbon dioxide		−0.009 (0.007)	−1.286	0.198	−	−	−	No significant interaction
TP*Urea		−0.005 (0.010)	−0.500	0.617	−	−	−	No significant interaction

RERI, relative excess risk of interaction; AP, attributable proportion due to interaction; S, synergy index; “−” indicates no significant multiplicative interaction, and additive interaction analysis was not performed; AST, aspartate aminotransferase; TP, total protein.

### 3.6 Nomogram construction and comprehensive performance evaluation

**Figure 5:**
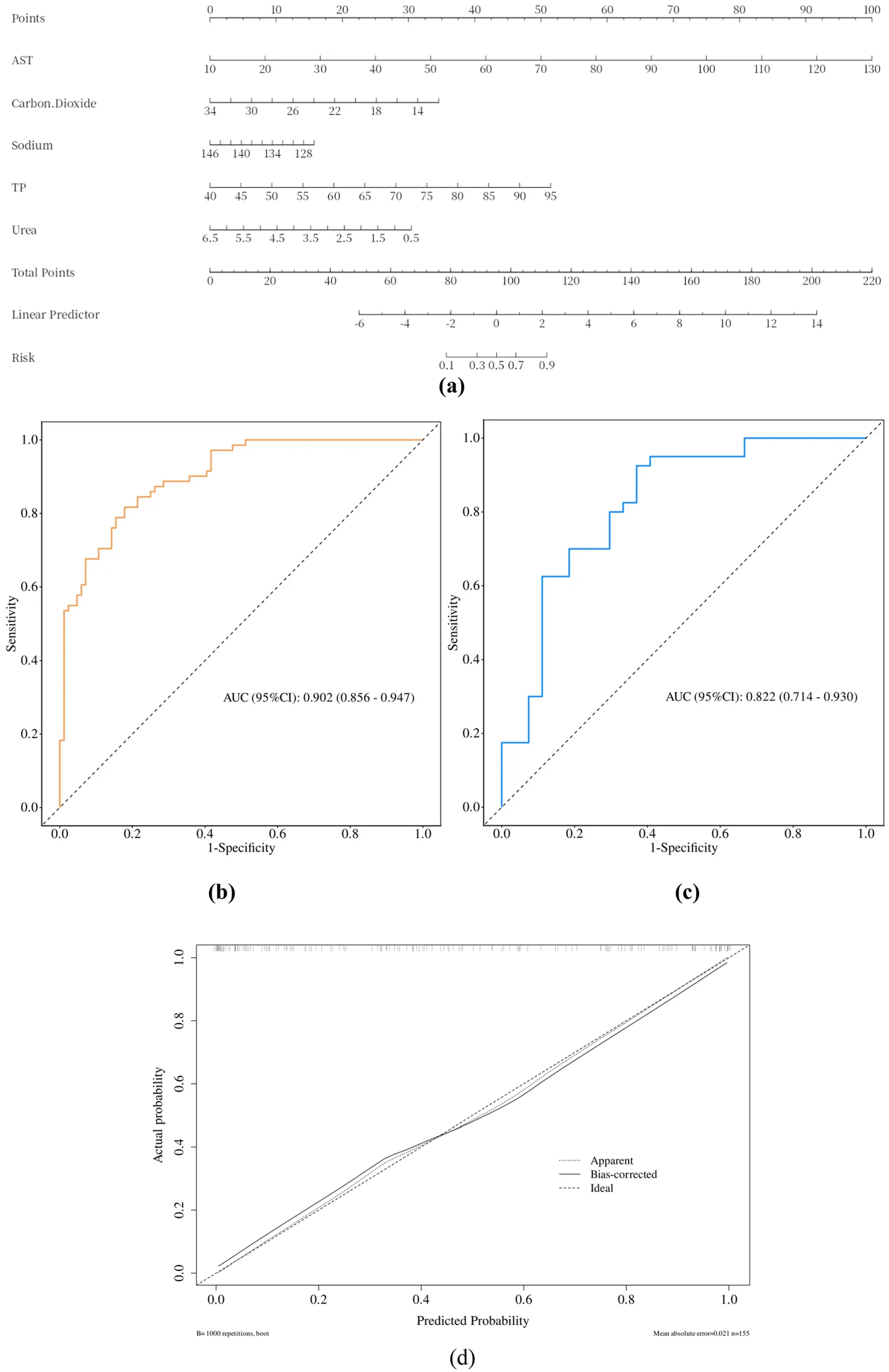
Nomogram model for predicting the risk of outcome events and its performance validation. **(a)** Nomogram for predicting the risk of outcome events constructed based on five independent influencing indicators; **(b)** Receiver operating characteristic (ROC) curve of the training set; **(c)** Receiver operating characteristic (ROC) curve of the validation set; **(d)** Calibration curve of the training set (1,000 bootstrap resamples); **(e)** Calibration curve of the validation set (1,000 bootstrap resamples); **(f)** Decision curve analysis (DCA) of the training set; **(g)** Decision curve analysis (DCA) of the validation set.

A risk-prediction nomogram was constructed based on the five independent predictors (AST, total protein, serum sodium, carbon dioxide combining power, and urea) derived from multivariate logistic regression. The point assignments for each predictor and the correspondence between total nomogram scores and the probability of myocardial injury are illustrated in Figure 5. The model exhibited excellent discriminatory performance, with receiver-operating-characteristic area-under-the-curve (AUC) values of 0.902 (95% CI: 0.856–0.947) in the training set and 0.822 (95% CI: 0.714–0.930) in the validation set. Calibration curves revealed favorable concordance between predicted and observed risks; the bootstrap-corrected mean absolute error was 0.021 for the training set and 0.035 for the validation set, indicating high predictive consistency. Decision-curve analysis (DCA) demonstrated that across a broad range of threshold probabilities, the nomogram yielded superior net clinical benefit compared with the “treat-all” and “treat-none” strategies, which supports its practical clinical utility.

**Figure:**
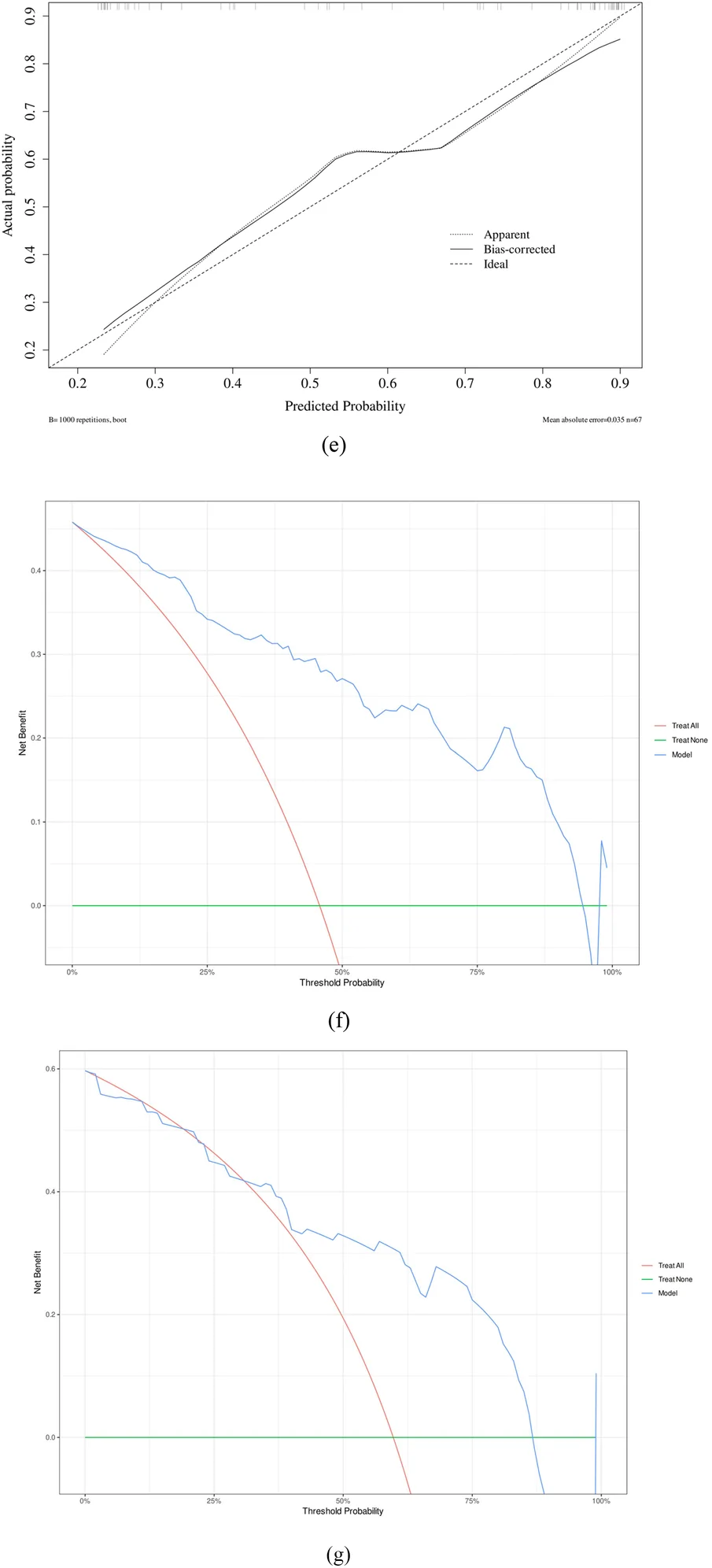


### 3.7 Subgroup stability analysis

Subgroup analyses stratified by age, sex, and disease course were performed to further evaluate the cross-population predictive performance of the established nomogram (Table 4). The model maintained robust discriminatory ability across all stratified subgroups: children aged ≤3 years (AUC = 0.868, 95% CI: 0.812–0.918, *P* < 0.001) and >3 years (AUC = 0.875, 95% CI: 0.821–0.922, *P* < 0.001); male patients (AUC = 0.870, 95% CI: 0.815–0.919, *P* < 0.001) and female patients (AUC = 0.873, 95% CI: 0.818–0.921, *P* < 0.001); and patients with a disease course ≤7 days (AUC = 0.866, 95% CI: 0.809–0.916, *P* < 0.001) and >7 days (AUC = 0.877, 95% CI: 0.823–0.924, *P* < 0.001). All subgroup AUC values were greater than 0.85, with no overlapping 95% confidence intervals. These findings confirmed stable and consistent predictive efficacy of the nomogram across different age groups, genders, and disease durations, demonstrating the model's excellent generalizability in clinical pediatric populations.

**Table 4:** Comparison of predictive performance among subgroup models.

Subgroup	AUC	95% CI	*P*-value
Infants ≤ 3 years	0.868	0.812–0.918	<0.001
Preschool and school-age children > 3 years	0.875	0.821–0.922	<0.001
Male	0.870	0.815–0.919	<0.001
Female	0.873	0.818–0.921	<0.001
Disease duration ≤ 7 days	0.866	0.809–0.916	<0.001
Disease duration > 7 days	0.877	0.823–0.924	<0.001

### 3.8 Development of a hierarchical risk stratification system

**Figure 6:**
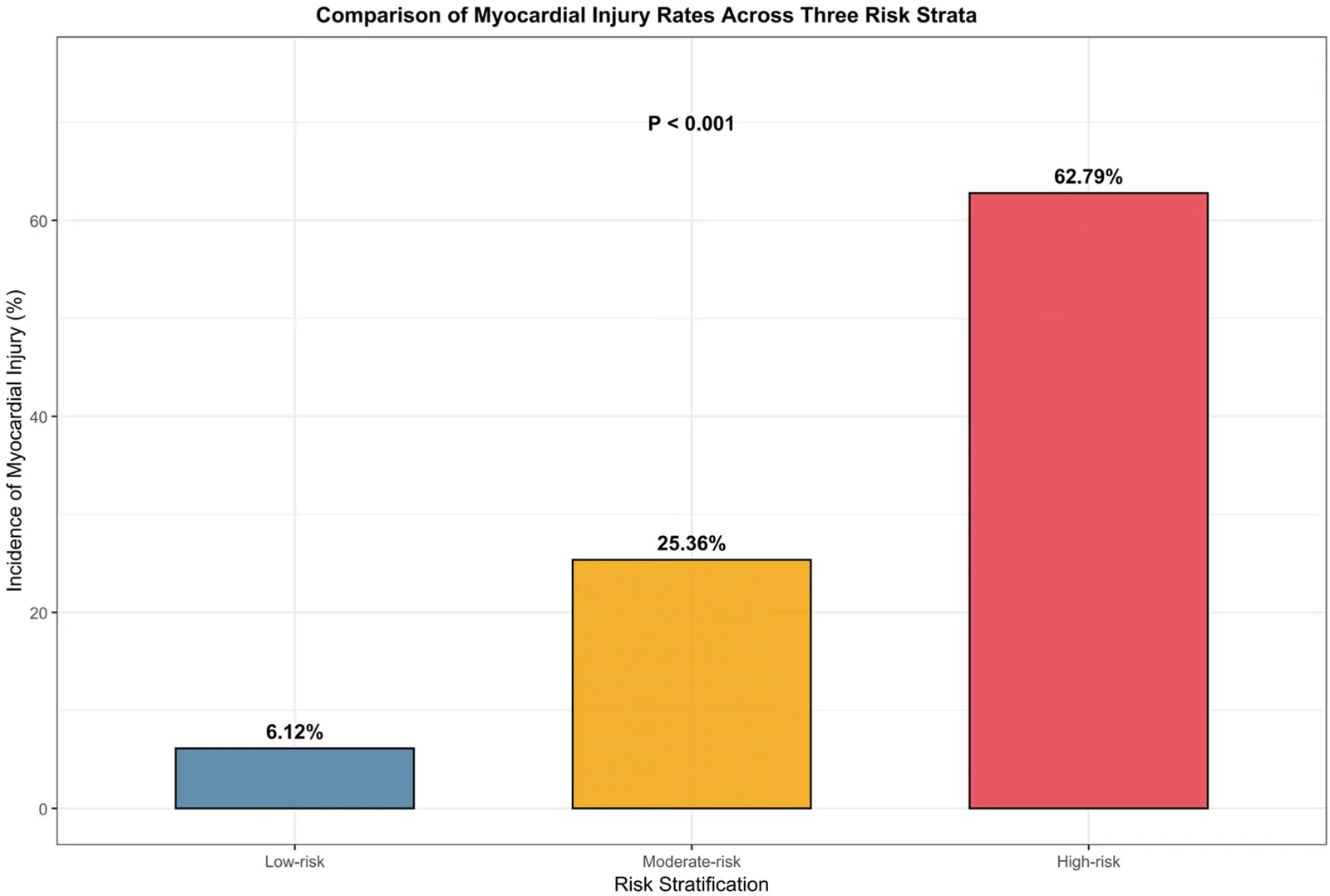
Bar chart comparing the incidence of myocardial injury across three-tier risk stratification. This figure visually presents the graded differences in the incidence of myocardial injury among the low-risk, medium-risk and high-risk groups, verifying the clinical discriminatory ability of the stratification system.

Based on the total scores derived from the nomogram model, a three-tiered hierarchical risk stratification system was established to enable precise clinical identification of high-risk children. In accordance with the distribution of predicted risks and clinical applicability, patients were classified into low-risk (score < 80), moderate-risk (score 80–160), and high-risk (score > 160) subgroups, with corresponding predicted myocardial injury probability thresholds of < 0.30, 0.30–0.70, and > 0.70, respectively. The actual observed incidence of myocardial injury varied substantially across the three strata: 6.12% in the low-risk group (76 patients), 25.36% in the moderate-risk group (104 patients), and 62.79% in the high-risk group (42 patients). The three risk gradients exhibited statistically significant differences in event rates (*P* < 0.001), verifying the favorable discriminatory performance of the stratification system. This objective grading framework can effectively distinguish pediatric patients with distinct myocardial injury risks, thereby guiding individualized clinical interventions, optimizing medical resource allocation, and facilitating targeted disease management (Figure 6).

## 4 Discussion

This study systematically developed and validated a novel individualized nomogram model for predicting myocardial injury in children with pneumonia by integrating LASSO regression, restricted cubic spline (RCS) nonlinear analysis, multivariate logistic regression, and SHapley Additive exPlanations (SHAP) interpretable machine learning. The present results identified AST and total protein (TP) as independent risk factors, whereas serum sodium, carbon dioxide combining power, and urea served as independent protective factors. The established nomogram exhibited excellent discrimination, favorable calibration, and robust clinical net benefit, with stable predictive efficacy across diverse patient subgroups. This validated quantitative tool provides clinicians with an objective, convenient approach for the early identification of pediatric patients at high risk of pneumonia-associated myocardial injury.

Univariate analysis demonstrated that children with myocardial injury were significantly younger and had lower body weight than those without myocardial injury (*P* = 0.003 and *P* = 0.009, respectively), indicating that younger and underweight pediatric patients are more vulnerable to secondary myocardial injury during pneumonia. This finding is consistent with existing evidence: immature cardiomyocytes in infants possess impaired sarcoplasmic reticulum calcium regulation and reduced tolerance to inflammation, hypoxia, and electrolyte disorders, rendering the immature myocardium more susceptible to damage under systemic infectious stress. Additionally, the incompletely developed immune system in young children contributes to uncontrolled pro-inflammatory cytokine release after infection, further exacerbating indirect inflammatory myocardial impairment (26). Notably, conventional inflammatory markers, including PCT, CRP, and WBC, were comparable between the two groups, suggesting that generalized inflammatory biomarkers lack specificity for predicting pneumonia-related myocardial injury. Instead, electrolyte disturbance, acid-base imbalance, and metabolic dysfunction better reflect the core pathophysiological characteristics of myocardial damage. These observations are consistent with the findings reported by Shi et al. (27) in children with severe pneumonia, which confirmed that metabolic and organ functional indicators outperform routine inflammatory markers in predicting extrapulmonary complications.

Multivariate regression and SHAP analyses consistently verified that AST is the most critical independent risk factor for myocardial injury (OR = 1.11, 95% CI: 1.07–1.15). RCS analysis further identified a significant nonlinear correlation, with a clear risk threshold of 45 U/L, above which myocardial injury risk increases progressively. AST is widely distributed in cardiomyocytes, hepatocytes, and skeletal muscle cells. Myocardial injury increases cell membrane permeability and elevates serum AST concentrations, which rises earlier than the highly specific cardiac troponin I, making AST a sensitive early biomarker for acute myocardial damage (28). Moreover, pneumonia-induced systemic inflammation and hypoxia can trigger secondary hepatic injury and further AST release. Subsequent hepatic dysfunction impairs the synthesis of coagulation factors and proteins as well as the clearance of toxic metabolites, thereby aggravating myocardial damage (29). As a routine, low-cost liver function indicator, AST exhibits excellent accessibility and broad application prospects, particularly in resource-limited primary clinical settings (30).

TP was also identified as an independent risk factor (OR = 1.12, 95% CI: 1.04–1.21), which appears counterintuitive and requires contextual pathophysiological interpretation. In acute infectious and inflammatory states, elevated serum TP primarily reflects compensatory hepatic synthesis and secretion of acute-phase proteins (e.g., CRP, fibrinogen, *α*1-antitrypsin), rather than improved nutritional status (31). Enhanced acute-phase protein synthesis mirrors the systemic inflammatory burden, which shares overlapping pathological mechanisms with myocardial inflammatory injury (32). Furthermore, elevated TP alters plasma osmotic pressure, disrupts myocardial cellular fluid balance and membrane potential stability, and indirectly promotes myocardial injury progression.

Serum sodium (OR = 0.81, 95% CI: 0.70–0.93), carbon dioxide combining power (OR = 0.86, 95% CI: 0.76–0.97), and urea (OR = 0.67, 95% CI: 0.45–0.99) were confirmed as independent protective factors, with decreased levels closely correlated with increased myocardial injury risk. These indicators reflect the stability of the internal environment, and their abnormalities imply disrupted homeostasis. Hyponatremia occurs in 15%–30% of children with pneumonia, mainly caused by inappropriate antidiuretic hormone secretion, fluid retention, and excessive sodium loss via respiratory and gastrointestinal pathways (33). Hyponatremia disrupts cellular osmotic equilibrium, induces cardiomyocyte edema and membrane potential instability, and ultimately leads to arrhythmia and decreased myocardial contractility. Multiple studies have validated hyponatremia as an independent predictor of adverse cardiac events in severe pediatric infectious diseases (34, 35). Reduced carbon dioxide combining power indicates metabolic acidosis or impaired acid-base compensation. Acidotic conditions inhibit myocardial Na⁺/Ca^2^⁺ exchanger activity and myosin ATPase function, suppress myocardial contractility, and activate cardiomyocyte apoptotic pathways, thereby exacerbating myocardial injury in critically ill children (36). The protective effect of urea does not indicate isolated renal function alteration but reflects altered protein catabolism and decreased renal tubular reabsorption under severe infection, representing reduced systemic metabolic reserve (37). The threshold values determined by RCS analysis (sodium: 138 mmol/L; carbon dioxide combining power: 22 mmol/L; urea: 3.4 mmol/L) provide practical quantitative targets for clinical internal environment intervention.

Interaction analysis revealed a significant additive synergistic effect between AST and TP (*S* = 1.32, 95% CI: 1.06–1.64), with their interaction contributing 16% of the total myocardial injury risk. The combined elevation of AST and TP produces a superimposed pathogenic effect that exceeds their individual risks, suggesting coordinated hepatic inflammatory injury and systemic acute-phase response jointly promote myocardial damage. This result supports the existence of a bidirectional “liver-heart axis” injury mechanism (38). In contrast, significant antagonistic interactions were observed between AST and sodium (*S* = 0.74) and between AST and carbon dioxide combining power (*S* = 0.78), indicating that maintaining electrolyte and acid-base homeostasis can effectively buffer the elevated myocardial injury risk induced by high AST levels. Clinically, this highlights a valuable therapeutic window: active correction of electrolyte and acid-base imbalances can mitigate myocardial injury even in patients with elevated AST. Compared with multiplicative interaction analysis, additive interaction quantification better explains etiological mechanisms and public health implications, which is more suitable for analyzing multifactorial complex diseases.

The newly constructed nomogram achieved favorable predictive performance, with AUC values of 0.902 and 0.822 in the training and validation cohorts, respectively, showing superior discriminative ability compared with previously reported predictive models for pediatric infectious disease complications (39, 40). The AUC difference of 0.080 between cohorts is within a reasonable range, verifying satisfactory generalizability. Bootstrap calibration with 1,000 resamples yielded mean absolute errors of 0.021 and 0.035 for the training and validation sets, both below the clinical threshold of 0.05, indicating high consistency between predicted and actual outcomes (41). Decision curve analysis (DCA) further demonstrated that the model provides sustained positive clinical net benefit across a wide range of threshold probabilities, confirming its reliable clinical applicability beyond statistical significance (42).

The integration of SHAP interpretable machine learning constitutes a major methodological innovation of this study. The ranked mean absolute SHAP values were highly consistent with multivariate regression results, and SHAP visualization intuitively quantified the magnitude and direction of each variable's predictive effect. This combined framework of traditional statistical regression and interpretable artificial intelligence compensates for the “black-box” limitation of conventional models, significantly improving the scientific credibility and clinical transparency of the prediction model (43). Integrating interpretable machine learning with clinical prediction modeling has been recognized as an advanced optimization strategy, and the present study extends this methodology to the field of pediatric infectious diseases (44).

Stratified subgroup analyses based on age, sex, and disease course showed that all subgroups maintained AUC values above 0.85 with non-overlapping 95% CIs. The stable predictive performance even in infants aged ≤3 years (AUC = 0.868) demonstrates robust cross-population validity and broad clinical applicability (18). Unlike previous models that often lose accuracy in younger infants, the present model performs reliably in low-age pediatric populations, further supporting its practical clinical value.

The three-tier risk stratification system established based on nomogram scores exhibited significant gradient differences in myocardial injury incidence: 6.12% for low-risk patients, 25.36% for moderate-risk patients, and 62.79% for high-risk patients (*P* < 0.001), forming an approximately tenfold risk difference across strata. This precise stratification strategy enables individualized clinical management. Low-risk children can receive routine pneumonia treatment with reduced unnecessary cardiac function monitoring; moderate-risk patients require intensified cardiac surveillance and selective imaging follow-up; high-risk patients need timely myocardial protection, active correction of internal environmental disorders, optimized anti-infective therapy, and early cardiology consultation. This quantitative “precision stratification and individualized intervention” model effectively avoids clinical overtreatment and undertreatment and facilitates rational allocation of medical resources (45).

This study has several limitations. First, the single-center retrospective design may limit the generalizability of the findings, and further multicenter prospective validation is required. Second, although internal bootstrap validation confirmed model stability, external independent validation is still lacking. Third, this study did not conduct pathogen subtyping analysis; therefore, the influence of bacterial vs. viral pneumonia on predictive efficacy remains unclear. Fourth, only admission static indicators were included, without dynamic monitoring of biomarker changes during disease progression, which may ignore valuable temporal variation characteristics. Future studies will focus on prospective multicenter cohorts and explore the incremental predictive value of longitudinal dynamic biomarker monitoring.

AST, TP, serum sodium, carbon dioxide combining power, and urea are independent influencing factors for myocardial injury in children with pneumonia, with distinct synergistic and antagonistic interactions among these indicators. AST exhibits a significant threshold effect on myocardial injury risk. The newly developed nomogram prediction model and three-tier risk stratification system possess stable predictive performance, convenient operability, and low detection costs, and are suitable for clinical promotion even in primary medical institutions. This tool enables early bedside screening of high-risk children, guides timely correction of electrolyte and acid-base disorders and organ function intervention, and ultimately helps reduce the incidence of pneumonia-associated myocardial injury and improve clinical prognosis in pediatric patients.

## Data Availability

The original contributions presented in the study are included in the article/Supplementary Material, further inquiries can be directed to the corresponding author.
